# Too clean, or not too clean: the Hygiene Hypothesis and home hygiene

**DOI:** 10.1111/j.1365-2222.2006.02463.x

**Published:** 2006-04

**Authors:** SF Bloomfield, R Stanwell-Smith, RWR Crevel, J Pickup

**Affiliations:** * London School of Hygiene and Tropical Medicine London, UK; † Royal Institute of Public Health London, UK; ‡ Safety and Environmental Assurance Centre Unilever, Bedford, UK; § Consultant in Scientific Issues Bridgnorth, Shropshire, UK

## Abstract

The ‘hygiene hypothesis’ as originally formulated by Strachan, proposes that a cause of the recent rapid rise in atopic disorders could be a lower incidence of infection in early childhood, transmitted by unhygienic contact with older siblings. Use of the term ‘hygiene hypothesis’ has led to several interpretations, some of which are not supported by a broader survey of the evidence. The increase in allergic disorders does not correlate with the decrease in infection with pathogenic organisms, nor can it be explained by changes in domestic hygiene. A consensus is beginning to develop round the view that more fundamental changes in lifestyle have led to decreased exposure to certain microbial or other species, such as helminths, that are important for the development of immunoregulatory mechanisms. Although this review concludes that the relationship of the hypothesis to hygiene practice is not proven, it lends strong support to initiatives seeking to improve hygiene practice. It would however be helpful if the hypothesis were renamed, e.g. as the ‘microbial exposure’ hypothesis, or ‘microbial deprivation’ hypothesis, as proposed for instance by Bjorksten. Avoiding the term ‘hygiene’ would help focus attention on determining the true impact of microbes on atopic diseases, while minimizing risks of discouraging good hygiene practice.

## Introduction

When a disease, or group of diseases, rises rapidly without a specific explanation, it stimulates investigation to identify the cause, so that preventive measures can be devised. An example of this is the rise in the incidence and prevalence of atopic disorders that has occurred over the last 30–40 years.

Although genetic predisposition is a fundamental factor governing susceptibility to atopic disease, the rise in atopy has occurred within too short a time frame to be explained by a genetic shift in the population, thus pointing to environmental or lifestyle changes. Although some increase in exposure to allergens to sensitize and then trigger attacks has been observed, such as increased exposure to house dust mites in modern, centrally heated houses, these changes do not seem adequately to explain the rise. This has led to the search for other factors to explain increased phenotypic expression of atopic disease in genetically predisposed individuals.

The ‘hygiene hypothesis’ was first formulated in 1989 by an epidemiologist, Dr Strachan [1] who reported an inverse relationship between family size and development of atopic disorders, and proposed that a lower incidence of infection in early childhood, transmitted by unhygienic contact with older siblings or acquired pre-natally could be a cause of the rise in allergic diseases. Subsequently, as the concept was further explored by specialists in allergy and immunology, it evolved into the broader notion that declining microbial exposure is a major causative factor in the increasing incidence of atopy in recent years. A wide range of factors which might have resulted in altered microbial exposure have been examined such as clean water and food, sanitation, antibiotics and vaccines, birth practices, as well as incidental factors such as the move from farm to urban living.

Most recently, a further aspect of Strachan's hypothesis has received considerable attention, particularly from the media, namely his proposition that the reason why this key exposure no longer occurs, or occurs to an insufficient extent, is the trend not only towards smaller family sizes but also ‘improved household amenities and higher standards of personal cleanliness’– in effect, cleaner homes.

Until recently, discussion of the hypothesis has been relatively compartmentalized, with most research focusing on the nature of the possible link between atopy and microbial exposure. Research into other factors that could explain the atopy trends, such as changes in diet and other shifts in lifestyle have continued in parallel. It is only recently that infectious disease (ID) specialists have entered the debate, concerned that publicizing the idea that we might be ‘too clean’ could have a detrimental impact on the public's perception of ID risks in the home and elsewhere, and of the importance of controlling such risks.

In response to these latter concerns the International Scientific Forum on Home Hygiene (www.ifh-homehygiene.org) commissioned a review of the hypothesis, in order to consider the implications it might have for hygiene, particularly hygiene in the domestic setting. It sought to do this by addressing two distinct questions:

- How clear is the evidence of a causal link between a decline in microbial exposure and the recent rises in atopic disease?
- To what extent might cleaning and hygiene, as distinct from other influences on microbial exposure, be a significant factor?

This review is a summary of the main findings from this report together with more recently published data [2].

### The rise in atopic disease

The rapid increase in allergic asthma and other atopic disorders in the industrialized world is usually considered to have started between 1960 and 1970 with progressive rises during the 1980s and ‘90 s, although Isolauri suggests that there has been a steady rise in sensitization to aeroallergens since the 1920s [3]. Asthma prevalence, for example, increased by about 1% a year from around 1980 [4]. Among children 5–18 years of age, the increase has predominantly been among allergic individuals [5] and recent UK studies confirm that atopic, rather than non-atopic, asthma is responsible for much of the rise [6, 7]. A comparison of two British birth cohorts (1958 and 1970) showed increases from 3.1% to 6.4% for eczema and from 12% to 23.3% for hayfever at age 16 years.

Both incidence and prevalence of atopy generally remain much lower in many, but not all the developing regions of the world. The International Study of Asthma and Allergies in Childhood (ISAAC) showed that the prevalence of self-reported asthma ranged from 2–3% in developing countries to 20–40% of the responding population of 13–14-year olds in industrialized countries, depending on the questionnaire used (written or video) [8]. The prevalence of other atopic disorders varied over a similarly wide range, with countries showing similar, but not identical rankings. Variations also appear between areas within countries.

The reunification of Germany provided some new insights into the influence of lifestyle on atopic disease within relatively homogeneous populations. Von Mutius et al. [9] showed that hayfever and atopic sensitization among children in the former East Germany both significantly increased between 1991–1992 and 1995–1996, raising the issue of ‘Western living’ influences on children, as previous studies had shown lower rates in East Germany compared with West Germany.

Some recent data suggests that the rise in incidence of atopic disease may be halting. A UK survey published in 2000 suggests that the number of new GP consultations for asthma is now falling, with declining reported episodes of asthma and acute bronchitis since 1993 [10]. Similarly, the third in a series of cross-sectional studies in Italy [11] showed prevalence to be stable among children born after 1985, in contrast to a threefold rise of prevalence between 1974 and 1992. The authors concluded that ‘the progressive induction of asthma symptoms in genetically predisposed subjects has probably come to an end’. Most recently, Z**ö**llner et al. [12] studying trends among children in SW Germany, have suggested the allergy epidemic may have reached a plateau.

### Evidence for and against a relationship between atopy and reduced microbial exposure

The proposition that reduced infection and/or changed microbial exposure may have driven the rapid rise in atopic disease requires evaluation on several levels. These include biological plausibility, the strength of the epidemiological data, the temporal correlation between trends in atopy and trends in ID, prevention measures and hygiene practice. Biological plausibility is one of the greatest strengths of the hygiene hypothesis. The hypothesis generally fits with current knowledge about maturation and disorders of immune system e.g. dysregulation of T helper type 1 (Th1), Th2 and regulatory T cell pathways, although the immunological models are constantly being revised and refined. It is not the aim of this paper to assess current immunological knowledge and closeness of fit with the hypothesis. Both have been reviewed elsewhere [13, 14] and, for the purpose of this review, biological plausibility is accepted.

Epidemiological studies of the relationship between prevalence of atopy and measures of infection and microbial exposure

The hygiene hypothesis originated not from observations about infection, but from data suggesting a relationship between atopy, family size and birth order. The link to infection was an assumption that these factors could be proxy measures of infection.

As the hypothesis has evolved, the range of potentially significant exposures under consideration has widened beyond those that result in clinical infection to include sub-clinical infection, colonization or even seemingly inconsequential exposure. Similarly, the range of microbes postulated as responsible has widened to include not only pathogens, capable of causing infection, but also non-pathogenic types or strains (commensals and environmental strains), and components of microbes such as bacterial endotoxins. There has also been a growing appreciation of the diversity of contributions by different microbes. Studies investigating the relationship between direct measures of infection or microbial exposure rates and atopy necessarily rely on epidemiological data. Even for confirmed clinical infection, accurate measures of infection rates are difficult to obtain retrospectively, especially for common, relatively minor, childhood infections. Surveillance systems are geared to monitoring diseases that are serious enough to be notifiable, and the vast majority of common infections go unrecorded. Measuring sub-clinical infection or the extent of colonization with environmental strains is even more difficult.

Many investigations have thus used other proxy measures of microbial exposure such as farm living, bed sharing and attendance at day nurseries. These studies will be reviewed first, before turning to studies that have examined more direct measures of microbial exposure and/or infection.

#### Family size and structure and atopy

Associations between atopy and family structure have been found in many studies, although the associations are less consistent for individual atopic diseases, and sub-divisions such as birth order, sibship size and gender.

An inverse association between atopy and family size was found in studies using hayfever, skin prick positivity and specific IgE levels as markers [15–17]. For asthma, Haby et al. [18] reported a protective effect of three or more older siblings on children aged 3–5 years, using a broad definition of asthma as a clinical diagnosis, cough or wheeze in the previous 12 months. A case–control study of clinically diagnosed asthma compared with healthy controls [19] also showed a small family size effect for cases diagnosed between 3 and 4 years of age.

The protective effect in large families was found to be stronger from older siblings [20] and from brothers compared with sisters [21, 22]. Bodner et al. [23], however, found that older siblings decreased the probability of hayfever and eczema, whereas the risk of asthma was reduced by the presence of younger siblings. Analysis of allergies among children of 11 042 women enrolled in the ALSPAC study [15] found a protective effect from brothers for inhalant allergy, but no significant trends for overall family size. Seaton and Devereux [24], using a cohort followed up since a primary school study in 1964, found membership of a large family reduced risks of hayfever and eczema, but offered no significant protection against asthma. Furthermore, the effect of a large family was not explained by infections the child had suffered: in contrast, the larger the number of infections, the greater the likelihood of later asthma, with the exception of a modest protective effect of measles. Genetic predisposition to allergy as indicated by an atopic parent also complicates the picture: Mattes et al. [25] found a sibship relationship only in children of atopic fathers, whereas in the European study by Svanes et al. [21], family size influence on prevalence of specific IgE was restricted to children with no parental history of allergy.

Differences related to gender and birth order thus remain unexplained. There is little temporal relationship between family size and the rise in atopy: the major decline in family size dates from the early 20th century in the UK and in most other industrialized countries where atopic disorders have increased. Wickens et al. [26] estimated that declining family size could account for only a small proportion of the rise in atopy between 1961 and 1991, for example only 1% of the rise in asthma and hayfever in England and Wales during this period.

The epidemiological studies show that although these factors are related to the incidence of atopic disease, they do not explain how the relationship operates. A wholly different explanation to the hygiene hypothesis has been suggested by workers focussing on changes in the mother, rather than in the environment of the offspring. Karmaus et al. [27] suggests that the sibling effect originates *in utero* and that ‘the negative association of infections and atopic manifestation is not causal but more likely to be spurious’. In a study of 981 newborn babies in the Isle of Wight, England, they found that levels of cord blood IgE reduce with increasing birth order. Subsequent studies [28] showed that maternal IgE decreases with birth order, and suggest that the decrease in cord serum is an indirect effect of the reduction in maternal IgE. This could explain why younger siblings have less later atopy. Similarly, Devereux et al. [29] compared Th cell proliferative responses in cord blood samples from a cohort of 2000 births, according to birth order, maternal smoking and maternal dietary intake. The magnitude of the Th cell responses to allergens decreased with birth order and high maternal vitamin E intake, but increased with a family history of atopy or maternal smoking. They conclude that birth order, diet and smoking are the risk factors in the maternal environment for subsequent atopy.

#### Other proxy measures and atopy: microbial exposures in households, day nurseries, rural environments, farms and places of work

(i) Close contact: bed sharing and day nurseries. In the European Community Respiratory Health Survey [21], sharing a bedroom as a child, a factor more likely in large families, had a protective effect on the subsequent risk of atopic disease. As Strachan [30] observes, this accords with the hygiene hypothesis, in providing more opportunity for exposure to microbes or infection.

Because of the likelihood of increased exposure, an association between infections such as the common cold and day care attendance would be expected. This has been confirmed, e.g. by the Tucson Children's Respiratory Study, a prospective study in large day care facilities in the USA [31]: the children had more frequent colds at year 2 and fewer colds at the ages 6–11, than those cared for at home. There was no evident protective effect against colds by the time they had reached 13 years of age, and no protective effect was observed for children in small day care facilities. Results are inconsistent for studies investigating whether early exposure to large day care facilities protects against atopic disease.

Some studies report reduced risk of atopy [32–34] while others show no protective effect [35, 19]. The effects on asthma and on other respiratory symptoms may be in different directions at different points in time. Using data from the Tucson Children's Respiratory Study, Ball et al. [31] reported that asthma was less frequent in children with one or more siblings at home or who had attended day care during the first 6 months of life. However, children with more exposure to other children at home or in day care were more likely to have frequent wheezing at the age of two, but had a decreased risk from the age of 6–13 years. Increased risk of transient childhood asthma associated with day care attendance was reported for Canadian children [19], although factors such as short duration of breastfeeding contributed to the raised risk; and the risk was decreased for persistent cases (6-year follow-up) with a history of day care attendance.

(ii) Farm and other rural exposure. Differences between people living in the towns or the country have long aroused curiosity. Recent studies have isolated the farm, rather than the general rural environment as the factor conveying a protective effect against atopy. For example, several studies report a reduced incidence of hayfever in children of farmers, as compared with other rural dwellers [36–39].

In the 16-year serological and questionnaire survey reported by Gassner-Bachman and Wuthrich [37], a statistically significant increase in the incidence and severity of hayfever and asthma, and of sero-prevalence of sensitization, was found in rural children with no direct contact with agriculture. By contrast, farming children had less atopic disease and lower levels of seroprevalence to a wide range of allergens, including those to which they had high exposure. An inverse dose–response relationship was identified, with children who had intermittent farm contact showing intermediate results.

Several factors have been associated with the lower prevalence of some allergies but not others: contact with animals as a child, exposure to stables under the age of 1 year, and consumption of farm milk (presumably raw/ unpasteurized) [38–40]. However the specific exposure has not been fully characterized. Much recent interest has focussed more specifically on bacterial endotoxin as the possible protective factor.

Most of the farm studies rely on serological measures of atopy or on skin prick tests (SPTs): the recent results from the ISAAC study [8] suggest that this may be an unreliable way of testing for differences in atopy and asthma between population groups. Although Leynaert et al. [41] found adults who had lived on a farm as a child had lower levels of cat sensitization and a lower risk of nasal symptoms in the presence of pollen, they found no consistent association between living on a farm in childhood and the risk of asthma or wheeze: a similar proportion had ‘atopic asthma’ in the farm and non-farm groups.

There is, however, no epidemiological evidence that working on a farm *per se* is associated with reduced risk of adult atopy. All the available evidence about the protective effects of exposure to farm environments discussed above relates to early childhood exposure. Farmers have a higher risk of occupational allergic disease and long-term exposure to endotoxins can harm lung function [42]. Similar hazards (but no evidence of protection against atopy) are reported for waste disposal and sewage workers [43].

#### More direct measures of microbial exposure and/or infection

Despite the difficulties outlined previously, several studies have attempted to evaluate the link between atopy and more direct markers of microbial exposure and/or infection.

Food-borne and gastrointestinal disease:

- Bacterial and viral infections. The most consistent evidence for an inverse relationship between exposure to a specific pathogen and atopy is shown by Hepatitis A virus (HAV), an infection associated with large family size and low socio-economic status [30]. Matricardi et al. [44] found that the prevalence of raised aeroallergen-specific IgE was halved in Italian military students with evidence of previous HAV infection. This finding was independent of age, sibship size, birth order, area of residence and parental education. Matricardi et al. [45] and Bodner et al. [46] also found seropositivity for HAV in the general population was associated with 40% and 37% reductions in atopy, respectively.
  A retrospective case–control study in Italy among male air force cadets by Matricardi et al. [47], found that cadets with atopy had significantly lower serum levels than non-atopic controls of antibodies to *Toxoplasma gondii, Helicobacter pylori*, as well as HAV, although independent effects of particular infections were not assessed. The investigators concluded that early exposure to microbes via orofaecal and food-borne routes protect against respiratory allergy and that ‘hygiene and a westernized, semi-sterile diet may facilitate atopy by influencing the overall pattern of commensals and pathogens that stimulate the gut-associated lymphoid tissue’. However, some populations show no association between HAV or *H. pylori* status and atopic disease [48, 49].
  An important question is whether HAV acts at a molecular level, or is merely a marker for poor orofaecal hygiene and exposure to other gastrointestinal organisms. McIntire et al. [50] showed that infection by HAV may have a specific protective effect from atopy for individuals who carry a particular variant of the gene that encodes TIM-1 (also known as HAVcr-1), the cell-surface receptor used by HAV to infect human cells. In contrast, Matricardi emphasizes the importance of general (i.e. non-specific) exposure to infections during the ‘window’ period of immune system development in early life [51, 52].
  Studies of Sardinian children found that children hospitalized with non-typhoid salmonellosis were less likely to develop hayfever or asthma than children hospitalized with non-bacterial enteritis and the authors speculate that clinical or sub-clinical infection by *Salmonella* may contribute to the atopy protective effect of the farming environment [53].
- Endotoxin exposure. Several recent attempts to pinpoint the important underlying difference between farming and urban environments have focused on endotoxin exposure [39, 54]. Whether the mechanism is aerosolized exposure, for example, from animal dung, or increased exposure to Gram-negative organisms and their products in food, is not clear. In Southern Germany and Switzerland, von Mutius et al. [55] found that endotoxin concentrations were significantly higher in dust from kitchen floors and children's mattresses in farming than in non-farming families. This prompted a larger study in Germany, Austria and Switzerland [36] that found an inverse relationship between endotoxin in mattress dust and occurrence of hayfever, atopic asthma and atopic sensitization.
  Other studies looking at endotoxin variations in non-farm environments, [56] have found that higher levels of endotoxin in house dust are associated with less allergen sensitization in infants aged 9–24 months, as measured by skin prick testing. Böttcher [57] found endotoxin levels were higher in house dust in Estonia, where there is a low prevalence of allergy, than in Sweden, where allergy is high. The levels were inversely related to the development of atopic disease during the first 2 years of life in Swedish, but not in Estonian, children.
  Other workers [58, 59] however, have sounded notes of caution about the potential role of endotoxins in immune priming, not least about whether endotoxin *per se* is implicated or whether it is just a convenient, measurable marker for some other microbiological agent.
- Gut flora. One putative pathway for a protective effect of microbial exposure against atopy involves the bacterial flora of the gut. Bjorksten et al. [60] found that the intestinal flora of allergic children differed from those with no allergies: aerobic bacteria, coliforms and *Staphylococcus aureus* were more common in the flora of allergic children. The non-allergic children had a greater prevalence of *Lactobacilli* and *Bifidobacteria* spp. in their gut flora. In another comparison between Sweden and Estonia, Sepp et al. [61] found that Lactobacilli and Eubacteria were more common in Estonian infants. Bennett et al. [62] studied Ethiopian and Swedish neonates, finding that differences at birth were lost by 2 weeks of age, except for a persistence of Lactobacilli in Ethiopian infants. Adlerberth et al. [63] found a more rapid colonization rate and greater heterogeneity of intestinal microflora in Pakistani infants, compared with those in Sweden.
  Strachan [30] suggests that the key effect of farm living may be early programming of intestinal microflora, and several authors have suggested that endotoxins may have a ‘gut priming’ role.
  It has been suggested that ‘probiotics’ preparations commonly comprising lactic acid bacteria, such as *Lactobacillus* or *Bifidobacterium* may help prime or maintain normal gut flora and preserve intestinal mucosal integrity, which in turn may be beneficial to atopy and other immune-related conditions, including the response to infection. Probiotics may be protective against eczema more than other forms of atopic disorder [64]. Recent studies [65–68] suggest that probiotics may have a role in preventing respiratory and diarrhoeal diseases in children in situations where they are at increased risk of infection, e.g in day care facilities.
- Intestinal parasites. Various studies have suggested that intestinal infestation with helminths, particularly heavy and chronic infection, protects against atopy [69]. Self-reported wheeze was found to be lower in rural subsistence areas of Ethiopia than in the urban population [70]: a follow up study in the same population suggested that high degrees of parasite infection, particularly hookworm, prevented asthma symptoms in atopic individuals [71]. These findings are at first surprising as helminth infections promote a Th2 bias, and they were first explained by a ‘blocking’ mechanism whereby available cellular receptors for IgE were saturated with polyclonal IgE antibodies [69]. Helminth infections are also immuno-suppressive, however, and more recent explanations of the protective effect propose that this stems from promotion of a strong immunoregulatory network and anti-inflammatory effects that suppress symptoms in individuals otherwise predisposed to allergy [69, 72]. Other data, however, paint a more complex picture, indicate a suppressive effect on SPT reactivity, but not asthma or allergic rhinitis [73].

Respiratory and other non-gastrointestinal diseases and atopy:

- Measles. An inverse relationship between wild measles infection and atopy has been cited as evidence for the hygiene hypothesis, most particularly the evidence from a longitudinal study of children in Guinea-Bissau following an epidemic of wild measles [74]. An important feature of this ‘natural experiment’ was the high child fatality rate (25%) introducing possible survivor bias to the results. In contrast, no evidence of a protective effect from wild measles infection was found in the 1970 UK birth cohort [75], or in a Finnish survey of children exposed to measles in the early 1980s [76]. Although immunological mechanisms by which measles might protect against atopy can be postulated, the totality of epidemiological evidence is conflicting, and there is some evidence that measles is more likely to be harmful than protective in terms of the risk of atopic disorders [77].
- Mycobacteria. Mycobacteria are widely distributed in the environment, including commensal, opportunist and pathogenic strains, the latter causing tuberculosis in humans and animals. These microorganisms initially became of interest because of their ability to elicit strong protective Th1 immune responses.
  A study of 867 children in Japan aged 6 or 12 years at the time of routine tuberculin tests prior to BCG vaccination [78] suggested a protective effect of natural exposure to *Mycobacterium tuberculosis*. Others, however, have suggested this may reflect host effects rather than any causal effect between infection and atopy [79]. Alternatively, the benefits may be restricted to healthy subjects who do not develop tuberculous disease. Other retrospective analyses of BCG immunization programmes in relation to atopy have produced conflicting results [80–82].
  In a study in which tuberculosis notification rates in different countries were matched to ISAAC data on the prevalence of atopic symptoms von Mutius et al. [55] concluded that there is a trend to less atopy in countries with high TB prevalence, although this again could simply reflect the theory that the healthy individuals in those communities have enhanced Th1 responses and consequently less atopy.
  Mycobacteria may be of particular relevance to the hygiene hypothesis, since it has been suggested that early exposure to mycobacteria with low or no pathogenicity may protect against later atopic disease [83, 84]. The authors postulate that mycobacteria have a key role in ensuring proper regulation of the immune system by acting as stimulators for induction of regulatory T cells. They suggest that exposure to mainly innocuous mycobacteria in soil and water has been greatly reduced by water treatment and sanitation in Western urban environments.
- Respiratory viruses. Infection with *Respiratory syncytial virus* (RSV), rose during the 1980s, but RSV is mostly implicated in triggering asthma rather than protecting against it [85–87]. Children with atopy, particularly where there is a family history, may be more susceptible to its effects [88].
  Investigators are now interested in the possibility that RSV may be an important primer of the immune system during the second half of pregnancy or in early infancy [89], although this is based on immunological mechanisms rather than epidemiological studies. Similarly, although early evidence suggested that respiratory infections could exacerbate existing asthma, recent studies have focused on the possibility that viral respiratory infections could have a protective or immune system ‘priming’ role [90–92, 39].
- Malaria. A recent study in Gabon found that children with a positive skin test for atopy had a history of less infections and lower incidence of malaria than children who tested negative [93]. While these results could help to explain the lower prevalence of most forms of atopy in developing countries, this could also be a survival effect whereby children who survive malaria may have less genetic susceptibility both to infection and to atopy.
- Total burden of infection/microbial exposure. Some workers now favour the concept, not least as a solution to inconsistencies in the hygiene hypothesis, that the overall load of pathogenic/non-pathogenic microorganisms or high microbial turnover, rather than a specific type or types of microbes, is the key to immune system priming and reduced susceptibility to atopy [94–96, 52].
  A recent cohort study [92] produced evidence of a protective relationship: the total burden of infections was inversely related to subsequent development of asthma, with specific associations for repeated runny nose and herpes-like infections, but not bacterial, fungal or gastrointestinal infections. Matricardi and Bonini [52] found that the protective effect was confined to infections other than those affecting the lower respiratory tract. Conversely, other studies point to an increased rather than reduced risk of atopy following a combination of early infections [24, 97].

The temporal relationship between the rise in atopy and trends in infectious disease

If the trends in atopy are related to infection then, unless the mechanisms involved defer effects to later generations, the data should show a reduction in the incidence of ID just prior to the start of the rapid rise in the incidence of atopy in the 1960s/1970s. Infections should also have continued to decline during the first couple of decades, at least, of the observed rise. Also, if the window of opportunity for immune system ‘priming’ is pregnancy and early infancy, there should be evidence of a dramatic decline in ID 3–6 years before the steep rise in atopy.

The assumption that there has been a decline in ID in recent decades in countries with a notable rise in atopic disease, such as the UK, is understandable since the majority of data on ID trends derives from national surveillance data, the decline in hospital admissions for infection and the success of vaccination programmes and antibiotics. However, these data focus on serious, systemic infection and infection mortality, while the continuing burden of general ID, particularly sporadic or non-notifiable cases, tends to be overlooked. Even more neglected is the proportion of ID acquired in the home environment.

The decline in serious infections such as cholera and typhoid, associated with improvements in water, sanitation and personal hygiene, occurred in the late 19th and early 20th century in industrialized countries, much too early to be associated with the rise in atopy. For other serious notifiable disease, the decline in specific infections such as measles, mumps, rheumatic fever, tuberculosis and HAV dates from the 1940s onwards following the introduction of antibiotics and a comprehensive range of vaccines. These infections were cited recently as showing an inverse relationship with the incidence of immune disorders [98]. Yet in the UK, a country with high incidence and prevalence of atopic disorders and one of those showing the rapid post-1970 rise, there is no convincing relationship between trends in atopy and of infections as reported to national surveillance. The decline in TB started much earlier, and has reversed recently in London and other metropolitan areas. The decline in measles in the UK dates from the introduction of the combined measles, mumps and rubella vaccine in 1988. Similarly, a decline in HAV since 1994 in the UK following the introduction of an effective vaccine post-dates the rise in atopic disease. The longer term decline in HAV since the 1940s also shows no convincing temporal relationship with trends in atopic disorders.

Data indicate that, during the critical period relating to the rise in atopy, there has been a rise rather than a decline in many gastrointestinal infections. For example, food poisoning notifications increased from the early 1970s. Salmonella infections in England and Wales reached a peak in 1997. Although overall food poisoning figures for Salmonella are now declining, the commonest cause of bacterial gastrointestinal infection, *Campylobacter*, shows a continuing rise [99].

A study by Wheeler et al in 1995–6 confirmed the long accepted view that that national surveillance significantly underestimates the true incidence of infectious intestinal disease (IID) and revealed the extent of that underestimate for England and Wales [100]. This showed that many minor cases are not reported to general practitioners, and that the true figure is nearer to 9.5 million cases, suggesting that one fifth of this population suffer from one episode of IID every year. According to the UK Food Standards Agency about 50% of these infections are foodborne, the remainder being due to person to person transmission. Since many cases are ‘sporadic’ (not part of a recognized outbreak), and are not picked up by surveillance, it is reasonable to assume that a significant proportion of these infections occur in the community and in the home. The most recent WHO survey [99] suggests that about 40% of all foodborne infections across the WHO European region are caused by consumption of food in the home. The general trend for several IIDs such as rotavirus and norovirus infections is an increase over the past 20 years [101]. Wheeler [100] indicated that for every one case of rotavirus and norovirus reported to surveillance, at least 35 and 1562 additional cases respectively go unreported in the community.

Respiratory tract infections have also shown no overall decline, with the exception of those controllable by immunization, such as measles. The true incidence of respiratory tract infections is also likely to be much higher than the relatively few infections ascertained in laboratory reporting systems or by statutory notification. This particularly applies to the milder types of upper respiratory tract infection, such as the common cold and influenza-like illness. Consultation rates for influenza and influenza-like illness are well documented in the UK: a peak was noted in the winter of 1998–1999, of 200–400 per 100 000 population [99]: although influenza epidemics show a cyclic pattern and vary in severity, and thus also in the level detected by surveillance, there was no overall change preceding or during the rapid rise of atopic disorders. Similar patterns of influenza have been reported from other European countries. While RSV has declined slightly since 1997 [102], there was no recorded decline during the rapid rise of atopic disorders. For the common cold, there is no evidence of any change in incidence in recent decades or indeed over the last century. The evidence suggesting differences in frequency of colds and other minor infections related to day nursery attendance and other exposures, as reviewed earlier, may indicate differences in susceptibility between population subgroups, but it cannot be used to infer any overall increase or decrease in these infections in the population.

Although it is generally accepted that, in the US and Europe, helminth infections have steadily declined [103] there is no data available to show whether there might be a temporal trend suggesting a link between reductions in helminth infections and the rise in atopy in the developed world. However Asato et al reported rapid reductions in *Ascariasis* and *Trichuriasis* and hook worm infections during the 1950s and 60s, because of improved sanitation and improved living standards [104].

The relationship between the rise in atopy and measures taken to prevent and control infectious disease

A wide range of public health, medical and other changes have occurred over the past century such as clean water and food, sanitation, antibiotics and vaccines, all of which are likely to have resulted in significant alterations in microbial exposure and infection in the community:

#### Sanitation, water treatment and food quality

The evidence gives no support for a relationship between provision of treated water supplies and sanitation, and the rise in atopy over the last 30 years. Filtration and chlorination of municipal water dates from the late 19th and early 20th century in most developed countries: only a small proportion of consumers depended on untreated private water supplies after this era, particularly in the UK. Chlorination of mains water was introduced in 1908 in the USA [105] and 1910 in the UK [106]. Progress in sanitation followed the first connection of house drains and cesspools to sewers in 1848 in England. It was nearly fifty years before such systems were common. While the ‘sanitation revolution’ continued throughout the first part of the 20th century in developed countries, there appears to be no temporal association with the rapid rise in atopy over the last 30 years.

Changes in food and water quality designed to reduce our exposure to pathogens are likely to have altered our exposure to commensal and environmental strains as well as pathogens; removal of pathogens in modern water treatment inevitably also removes most benign microbial bacterial contamination, such as environmental mycobacteria. However, there is no direct temporal relationship with the rapid rise in atopy. Similarly changes in food preferences are likely to have altered the microbial content of our diet. Since foods are only controlled for pathogens, there are no available data to indicate what trends might have occurred in the broad microbial content of our diet during the period critical for the rise in atopy.

#### Immunization/vaccination

As vaccination involves the administration of attenuated or killed microorganisms, or selected components from them, in order to induce an immune response, the hygiene hypothesis predicts that it should influence susceptibility to atopic disease. However, epidemiological studies provide no consistent support for either a beneficial or adverse effect of vaccination/immunization on atopic tendency. The Guinea-Bissau study by Shaheen [74] suggested measles vaccination might increase the tendency to atopy, but this finding is not supported by other epidemiological studies. In a trial of pertussis vaccine in Sweden, Henderson [107] and Nilsson [108] found no convincing evidence of an effect on atopy.

Hurwitz [109] reported a twofold increase in asthma in a US study comparing vaccinated with unvaccinated children, but various authors have cautioned that, in countries with high vaccine coverage, families who choose not to immunize their children are unusual and that both increased risk of atopy and a protective effect could be demonstrated, depending on the population examined [110, 30]. In contrast, in a carefully controlled study, Ring and colleagues [111] found that former East Germans, whose take-up of pertussis vaccination was high, experienced lower rates of asthma than their West German counterparts, many of whom shunned this vaccine.

Although Rook and Stanford [83] suggested that early exposure to mycobacteria with low or no pathogenicity may protect against later atopic disease, several studies have reported a protective effect against immune disorders for BCG [82, 112–114]. The most supportive evidence comes from animal studies such as those showing suppression of allergen-induced eosinophilia in mice by infection with *Mycobacterium bovis* [115] and from promising initial results of trials with mycobacterial vaccines for the treatment of some diseases associated with immune dysregulation [116, 117].

#### Antibiotic therapy

The possibility of a relationship between antibiotic use and later asthma or other atopic disease is difficult to disentangle from the potential confounders such as whether the key exposure relates to the infection or the antibiotic [118]. A study in Germany by von Mutius [119] showed that six or more courses of antibiotics in the first year of life were associated with a later excess of hayfever and eczema, but SPTs did not indicate an increase in the prevalence of atopy. Similarly, a study in UK [120] reported that any course of an antibiotic before the age of 2 years was associated with a doubling of the risk of hayfever and eczema, particularly if the antibiotics contained cephalosporins and macrolides.

Bjőrkstén [121] has proposed that the antibiotic effect could be linked to the influence on the bacterial colonization of the gut in early years of development. In line with this a study with laboratory mice [122] showed that antibiotic-induced changes in the gastrointestinal tract can affect how the immune system responds to common allergens in the lungs. A recent study presented at the 2003 European Respiratory Society conference by Christine Cole Johnson, of the Henry Ford Hospital, Detroit, US (unpublished) showed that children who received antibiotics within their first 6 months of birth were up to three times more likely to develop allergies to pets ragweed, grass and dust mites by age 7. Of the 448 children studied, 49% had received antibiotics in the first 6 months of life. However, recent analysis of the relationship between antibiotics sales and the prevalence of symptoms of asthma, allergic rhinoconjunctivitis, and atopic eczema in 99 centres from 28 countries [123] found there was a positive association between per capita antibiotics sales and the prevalence of symptoms for asthma, rhinitis, and eczema, but the associations generally became negative once the analyses had been adjusted for GNP. Another recent study [124] alternatively suggests that, rather than antibiotic use in early life being associated with subsequent development of asthma, frequent antibiotic use in early life is more common amongst asthmatic children.

#### Breastfeeding

The well-established protective effect of breastfeeding against infection is mediated by transfer of maternal antibodies and by constituents affecting the infant's gut. Breastfeeding protects against intestinal pathogens [125, 126] and respiratory infections [127, 128], and it is somewhat paradoxical, therefore, that in some studies the reported benefits of breastfeeding include prevention of later asthma or atopic disorders [18, 129–132]. Breastfeeding may, however, also transfer active maternal viral infections such as HIV and Hepatitis [133].

The duration of breastfeeding appears to be an important factor, with little or no protective effect against atopy for short periods of breastfeeding, e.g. less than 3 months [134, 74, 135]. A dose-response effect was indicated in a retrospective longitudinal study of Canadian children aged 1–2 years: a higher risk of asthma occurred in children breastfed for up to 9 months, than in those breastfed for longer. The trend to shorter periods of breastfeeding, was also indicated in this study: 44% of the children were breastfed for only 2 months.

Auto-immune and other immune-related diseases

Several workers have looked for possible links between reduced microbial exposure and rises in certain auto-immune and other immune-related diseases which have accompanied the rise in atopic disorders. A variety of studies have found positive associations between various potential indicators of microbial exposure and inflammatory disorders regarded as Th1 mediated such as childhood diabetes [136–141], juvenile arthritis [142], Crohn's disease [143] and with childhood leukaemia [144–146]. However, as in the case of atopic disease, other studies find no association with these indicators [147–151, 144], while others suggest that certain infections may cause the disease [151–155].

### Other explanations for the rise in atopy, not linked to microbial exposure

The epidemiological evidence supporting the hygiene hypothesis as an explanation for the recent rapid rise in atopic disorders needs to be viewed in the context of other possible explanations. Indeed, it seems likely that the rise results from several factors, either operating independently, or perhaps with some measure of synergy. The observation that atopy is more prevalent among higher socio-economic groups has been cited as evidence for the hygiene hypothesis [30], although it could equally be a proxy measure for other, non-microbial factors, such as diet. Poverty has also been identified as a risk factor for asthma and other atopic disease [5, 156], and studies in the inner cities of the USA [157] suggest that the previously observed socio-economic gradient may be levelling out.

The effects of increased contact with house-dust mites, cockroach allergen, pollen and fungal spores (for example in damp housing) are primarily seen as triggering attacks or exacerbating symptoms of atopic disease rather than increasing susceptibility to atopy [158]. However, some studies show correlation between exposure to fungi for example and sensitization, positive skin-prick tests and IgE antibodies [159, 160]. The current balance of opinion about the effects of pollutants is similar, although perhaps moving further towards identifying situations where effects of exposure may go beyond triggering and exacerbation. For example, traffic emissions were reported to increase atopic sensitization for nine-year-old children living near major roads and in suburban areas in West Germany [161]. Ozone in urban air has been implicated as associated with increased risk of asthma [162] and heavy exercise in high ozone environments has been found to increase the asthma risk in children with no history of the disease [163].

One area of possibly more fundamental predisposition to atopy is that of nutrition and diet. Observed correlations between birth head circumference (which reflects increased birth weight [164] and raised IgE levels [165] have given rise to the suggestion that increased fetal growth trajectory resulting from improved nutrition in pregnancy and infancy may be achieved at the expense of immune system development. It is possible that changes in diet could mediate an effect, through changes in gut flora. Attempts have been made to alter the gut flora in infants [166], where manipulation is much easier than in the adult [167].

Obesity, now a major problem in children in developed countries, may be another factor in the rise in atopy. The body mass index (BMI) is positively associated with the risk of adult-onset asthma [168]. In a prevalence study by von Mutius [169], increasing BMI correlated with an increased risk of childhood asthma and atopy and BMI remained significant after adjustment for other factors. The consistency of the relationship with obesity, the temporal association and the dose response curve has prompted the theory that obesity causes asthma [170]. This is not yet a widely held view, since the biological mechanisms remain unclear [171]. Platts-Mills et al. [5, 172] suggests that the key changed factor, related to obesity, is less exercise.

In a study in Saudi Arabia, Hijazi et al showed that diet was more directly implicated as a predisposing factor for atopy [173]. As in other changing societies, asthma has increased in urban areas of Saudi Arabia. Hijazi et al. showed that diet may be the most important change in such societies, and most likely to explain the recent trends in atopic disease. The dietary factors involved include eating at fast food outlets, as well as lower intake of milk, vegetables, fibre and foods rich in Vitamin E. Dietary vitamin E was reported to inhibit IgE responses to allergic stimuli in a random sample of 2633 adults [174]. In a study of adult-onset wheeze in Aberdeen, intakes of vitamin E and plasma levels of ascorbate (a measure of Vitamin C) were inversely related to adult wheeze in the manual social class and among current smokers [175].

Changing diet, as an explanation of trends in atopic disorders, may not only have a ‘microbial’ effect by altering the gut flora (see above) but also has biological plausibility via non-microbial mechanisms e.g. in terms of the nutrients needed for healthy immune system development, such as polyunsaturated fatty acids and antioxidants [173]. The dietary hypothesis has also been coupled with the notion that there has been a shift in the population susceptibility to atopic disease [176], possibly also linked to increasing population mixing and greater genetic diversity [177]. In an investigation of whether reduction in childhood infections or change in diet could explain increases in asthma and atopic disease, Seaton and Devereux [178] found that low intake of vitamin C was associated with a sevenfold increase in the risk of bronchial hyper-reactivity; by contrast, those with the lowest intake of saturated fats had a tenfold lower risk. In a separate study, the lowest intake of vitamin E was associated with a fivefold increase in adult-onset wheezy illness, while the lowest intake of vitamin C doubled the risk. Dietary intakes were confirmed by measurement of vitamins and triglycerides in plasma. Seaton and Devereux concluded that changes in the diet of pregnant women may have resulted in the birth of cohorts of children predisposed to atopy and asthma.

The possibility that maternal influences or changes may be important is reflected in several studies. For example, a greater risk of atopic disease has been reported in children of older mothers [179]. The risk of childhood asthma is also greater for infants with a maternal compared with a paternal history of asthma [180], indicating the possible importance of maternal imprinting (preferential expression of maternal genes in the fetus) [181, 182], as well as the influence of the maternal-fetal interface [171]. Grandmaternal, as well as maternal, smoking has also been found to be associated with increased asthma risk in children, suggesting influences may extend beyond the parental generation [183].

For the protective factors discussed previously, such as large family size, farm life or breastfeeding, an important issue to consider is whether it is greater infection and/or greater microbial exposure, or other related ‘non-microbial factors’ which are the key. Family size undoubtedly influences the potential for case-to-case spread of infection by both aerosol and other routes, but it cannot be assumed that a large family inevitably causes increased spread of infection, or poor hygienic conditions: much depends on socio-economic factors such as overcrowding, sharing a bedroom, bed-sharing or awareness of how to prevent infections. The protective factors in farm life could include private, often untreated, water supply and non-microbial factors such as diet, exercise and outdoor activity.

### Evidence for and against the proposition that the reduced microbial exposure critical for immune development relates to improved hygiene practice, household amenities and personal cleanliness

The second proposition of Strachan's original hypothesis was that the reduced microbial exposure causing the rise in atopy was due to ‘improved household amenities and higher standards of personal cleanliness’. A popular view is that the microbial or infection exposure required for the development of a balanced immune system has been reduced as a result of the more ‘rigorous’ cleaning and hygiene practices which we now use not only to counter our fear of exposure to ‘germs’ but also to create a more aesthetically pleasing dirt-free environment in which to live. Evidence in relation to this question derives from the following sources:

Epidemiological studies of the relationship between the proxy measures of cleanliness and hygiene and the rise in atopy

#### Hygiene practices in relation to atopy

In two recent studies, Sherriff and co-workers [184, 185] examined the link between ‘hygiene behaviour’ and atopy by devising hygiene scores for personal hygiene practices, and looking for associations with wheezing and atopic eczema. The data was derived from the Avon Longitudinal Study of Parents and Children (ALSPAC). In self-completed questionnaires for 10 970 children aged 15 months, parents were asked how often in a normal day they wiped the child's face and hands, whether hands were wiped before meals and how often the child was given a bath or shower. The cumulative hygiene score derived from these responses was examined statistically in relation to socio-economic and peri-natal factors. These data suggested a ‘cleanliness norm’ for the majority of the children comprising washing face and hands 3–4 times a day, hands cleaned before meals, and a daily bath or shower. The hygiene scores ranged from 2 (least hygienic) to 14 (most hygienic). A score of > 10 was independently associated with maternal smoking during pregnancy, low maternal educational achievement, living in local authority housing and increased use of chemical household products (the latter based on a score derived from reported use of disinfectant, bleach and aerosols). High scores were also associated with higher maternal parity (2 or more children), short duration or no breastfeeding, overcrowded accommodation and little contact with dogs or other furry pets. In their follow up questionnaire study asking about atopic symptoms, Sherriff [184] reported that high scores for children aged 15 months were independently associated with increased risk of wheezing and eczema when this group of children reached 30 and 42 months of age. Mothers with a history of eczema were less likely to have children with a high score than non-atopic mothers, suggesting that knowledge of factors that exacerbate eczema influenced frequency of child bathing: no such association was found for mothers with a history of asthma.

While the responders in this long-standing ALSPAC study would be accustomed to answering questions, the association of high scores with social disadvantage (low educational attainment and overcrowded living conditions) and maternal smoking is surprising and raises the possibility of an association between social disadvantage and a tendency to exaggerate about personal cleanliness, particularly where this relates to childcare. Alternatively, these social factors may influence hygiene practice: for example, in younger, poorer or less educated mothers. The study also contains no measures of microbial exposure or how effective individual hygiene practices may have been in terms of reducing microbial exposure. Some studies [186] report that both asthma prevalence and severity are associated with social disadvantage. The findings from the ALSPAC study may thus reflect a non-causal association between asthma and hygiene behaviour.

#### Use of household cleaning products and atopy

A comparison of soap and detergent consumption with data on prevalence of atopic disease [187] showed no evidence of a relationship; plots of per capita consumption of soap, detergents and cleaning products in 1994 for 12 European countries against reported prevalence of asthma, hayfever and eczema as reported in the ISAAC study [8] showed no correlation. Nor was any correlation apparent between individual diseases and individual product types such as fabric washing detergents, dishwashing detergents, toilet soaps and hard surface cleaners. Consumption of household bleach, a highly effective disinfectant that is widely used by consumers, varies greatly across Europe but again shows no correlation with prevalence of atopic disease. Bleach consumption per capita is highest in Spain and other countries of southern Europe, which have relatively low incidence of atopy, whereas in Scandinavia, where bleach use is limited, some 30 times lower per capita than in Spain, atopy rates are relatively high.

Temporal relationship between the rise in atopy and trends in hygiene practice

As far as personal and other hygiene practices in the home are concerned, widespread access to clean water, soap and chemicals to aid cleaning dates back, with only a few exceptions to the end of the 19th Century, and thus significantly predate the rise in atopy. During the last half century availability of household amenities and appliances and the range and effectiveness of household cleaning products has increased. Water use has increased in all industrialized countries, including use both for personal cleanliness and for kitchen appliances such as dishwashers and washing machines.

Although the discovery of the transmission of infection by microorganisms led to much greater emphasis on hygiene in the home, this occurred mainly around the turn of the 19th/20th century. Prior to the 20th century, home hygiene was largely focussed on food preparation and storage. During the first half of the 20th century there was increasing emphasis on cleanliness in the home, with advice on regularly cleaning walls, ceilings and other areas, partly prompted by the fear of infection before the antibiotic era. An increasing number of products and equipment were developed for home cleaning during the last century, but other social changes during the latter part of the century changed the approach to housework and its extent:

- Domestic help became less available and more expensive;
- Women increasingly worked outside the home and had less time for housework;
- Vaccination and antibiotic therapy for treatment of infectious enemies, such as diphtheria and typhoid fever reduced perception that hygiene was important.

These changes led to a more superficial approach to home cleaning, with speed and aesthetic factors more important than hygiene and disease prevention. The trend towards the modern pattern of frequent bathing and laundering in the USA and UK dates from 1890 to 1915. Soap manufacture in the USA more than doubled between 1904 (700 000 tonnes (8.4 kg per capita)) and 1919 (1 700 000 tonnes, 16 kg per capita) [188]. The increasing popularity of showers in the USA occurred between the 1940s and 1960s, with the proportion of American homes with bathtubs and/or showers increasing from 61% in 1940 to 87% in 1960 [188]. Similar rises in showers, although slightly later, have occurred in European countries but the rise in atopy occurred at much the same time throughout the industrialized world.

Many cleaning agents used since the 1960s/70s are formulations of chemicals that have been available for a considerable time, such as bleach or phenolic disinfectants. The first detergent powders were introduced at the beginning of the 20th century. Usage of soap, detergents and cleaning products has continued to rise over the last 50 years, although at a lower rate than observed in the first quarter of the 20th century.

The data in Table 1 suggests a steady rise in consumption of soaps and detergents to a peak in the late 80s, followed by a decline to 1994. However, changes in product classification as well as formulation distort the picture such that only broad assessments are possible. For example, the rapid growth of liquid fabric washing and rinse conditioning products in the 1980s meant that the total consumption of product increased, but not the ‘cleaning power’ deployed. Conversely, the introduction of more concentrated liquid and powder products in the late 80s and early 90s will have had the opposite effect^.^ Taking into account these formulation trends [187] it is estimated that the real increase in cleaning product usage across the 12 European countries over the 25 year period would have been of the order of 50%. Such changes are considerably smaller than the variation in usage between countries, which range from 8 kg/cap in Finland to 30 kg/cap in Spain, although different national formulation preferences again distort this picture.

**Table 1:** Per capita consumption (kg) of soaps and detergents

	1969	1977	1988	1994
Toilet soap	0.806	0.859	0.702	0.630
Hard surface cleaners	0.730	1.794	2.977	2.060
Scourers	1.029	0.942	0.668	0.490
Hard soap	1.188	0.746	0.445	0.370
Dishwashing detergents	1.183	2.272	3.794	3.120
Fabric washing detergents	6.957	7.951	8.376	8.890
Others (by difference)	1.807	3.496	5.756	3.040
Total soaps and detergents*	13.700	18.060	22.718	18.600

* Including fabric conditioning products which have no cleaning action. Source of data: Extracted from Statistical Tables published by AISE (Association Internationale de la Savonnerie, de la Detergence et des Produits d'Entretien) 49, Square Marie Louise, B-1000 Brussels.

With regard to temporal trends for particular product types, the greatest increases have been in use of dishwashing products and hard surface cleaners, although the rise in the latter was substantially offset by the decline of hard soap and scouring products previously used for this purpose. Detergents based on synthetic surfactants, rather than soap, came into general use in the 1950s, thus pre-dating the rise in atopy.

In assessing whether the altered microbial exposure which may be responsible for the current trends in atopy bears a relationship to changes in our cleaning and hygiene habits, it is also necessary to evaluate studies of the impact of hygiene on microbial exposure in the community, and the various case control studies etc. assessing the impact on infection rates. These are discussed in the next part of this section.

Hygiene practice and microbial exposure and infection in the home

#### Microbial contamination and infection in the home

If we compare our modern centrally-heated, double-glazed, sealed homes, with homes of the 1950s where coal fires, urban pollution and open windows meant that housewives waged a constant war against dust and dirt, it is easy to make the assumption that modern homes are ‘cleaner’. In the immediate post-war years, lack of resources meant that European homes were less well maintained; cracked tiles, damaged flooring etc. all added to giving homes the appearance of being ‘dirty’.

In recent years a range of studies have been published, as reviewed by Beumer et al. [189], showing that pathogenic, commensal and environmental microbial species are all introduced continually into the home by people, food, water, pets, and sometimes via the air, and that our modern home environments, despite their clean appearance still offer constant opportunities for microbial exposure.

As far as food is concerned, exposure to food-borne microorganisms (both pathogens and commensals) during food handling in the home must be a fairly common occurrence. Surveys of chickens in the UK between 1979 and 1998 indicated contamination rates between 25% and 79% for *Salmonella* [190] and 80–90% for *Campylobacter* [191, 192]. While recent data [193] suggest that these rates have now fallen, data on the high chicken consumption in the UK suggest that at least one in 25 UK homes prepare a meal with contaminated chicken every day of the year. Similar high rates of contamination are reported from other European countries, such as the Netherlands, France, Italy and Germany [194]. Cattle and sheep are important sources of *Escherichia coli* O157; Chapman et al. [195] showed that 0.4–0.8% of meat products purchased from UK butchers were positive for *E. coli* O157. Domestic cats and dogs in the home can act as reservoirs and shedders of *Salmonella, Campylobacter* and other enteric pathogens as well as commensal strains [196]. In a recent study [197], 19 species of *Campylobacter*, including *C. upsaliensis* and *C. jejuni* were isolated from 100 faecal specimens obtained from a London cattery.

Exposure to microorganisms from these human, animal, food and environmental sources can occur either by direct contact or by transfer involving inhalation of infected aerosols, consumption of contaminated food or water, or indirect transfer via hands or other surfaces into the nose, mouth or eyes, or into open cuts or wounds etc. Laboratory-based studies [198–203] show that bacteria and viruses spread to environmental surfaces from an infected or carrier source can survive in significant numbers for periods of several hours and in some cases days particularly on surfaces where moisture is present but also on dry surfaces. Other studies show that, when surfaces become contaminated, the organisms are readily spread via hands, cleaning cloths, and hand and food contact surfaces around the home, providing continual opportunities for human exposure [200, 204–206]. Using a bacteriophage φX174 as a model, Rheinbaben et al. [207] showed that, following contact (handshaking) with a volunteer whose hands were contaminated from touching a virus-contaminated door handle, successive transmission from one person to another could be followed up to the sixth person. Similarly, Cogan et al. [200] demonstrated that, following preparation of *Salmonella-* and *Campylobacter-*contaminated chickens in domestic kitchens, these species could be isolated from 17.3% of the hands, and hand and food contact surfaces sampled.

Although raw food is probably the main source of microbes in kitchens, there is evidence for a contributory role of surfaces such as draining boards, sinks, U-tubes, nappy buckets, dishcloths and cleaning utensils: wet sites, in particular, may act as permanent sources or reservoirs of free-living bacterial populations. In the bathroom or toilet, enteric bacteria probably originate from the toilet or directly from people, but permanent reservoirs of bacteria readily survive in baths, basins, cleaning cloths and face cloths [209]. Most species isolated in these studies are not normally pathogenic, but the evidence suggests an abundant population of microorganisms in the home. An evaluation of dust samples in 20 US homes showed high levels of endotoxin on kitchen and bedroom floors [208]. These and other data [209–212] confirm that all types of microbes, are found in all areas of the home environment, and that patterns or levels of microbial contamination have not significantly altered in the 20 years between the earliest and most recent of these studies.

The extent to which exposure to food-borne pathogens still occurs in the home is suggested by community-based [100] estimates that, each year, one in five of the population in England and Wales suffers a bout of IID. While contaminated food accounts for about 75% of reported infections attributed to bacteria, enteric viral infections are spread mainly by person-to-person contact or via aerosolized particles resulting from vomiting or fluid diarrhoea. During and after viral infections (both respiratory and enteric viral infections), virus particles are shed in large numbers in body fluids including blood, faeces, saliva and nasal secretions. There is relatively little data on the occurrence of viruses in the home environment, but a recent study in five US homes where there was an influenza-infected child showed the presence of influenza A virus on between 20% and 100% of the surfaces sampled, which included telephone receivers, door knobs, toilet handles and computer surfaces [213]. Other studies show that surface to finger/surface to mouth transmission of rotavirus readily occurs [214, 215]; in a laboratory study, Ward et al. [214] showed that 13 out of 14 adult subjects became infected after consuming Rotavirus (10^3^ particles). The potential for norovirus transmission from person-to-person via hands and surfaces is indicated by the recurrent infection outbreaks in successive cohorts of guests in hotels and cruise ships [216, 217]. Epidemiological studies suggest that, whereas aerosols are the main route of dissemination within a cohort of people, contaminated surfaces are responsible for ongoing outbreaks by forming the link between successive groups. A recent study [218] shows the ease of spread of norovirus via hands, cloths and other surfaces. There is now growing evidence that respiratory infections such as rhinovirus and RSV infections can also be spread via hands and surfaces such as handkerchiefs, tissues, door handles and telephones; indications are that the virus is transferred via the fingers to the nasal mucosa or conjunctiva; self-inoculation with rhinovirus by rubbing nasal mucosa or the eye can lead to infection [219, 220].

A key questions with regard to the hygiene hypothesis, which remains to be addressed, is how big the critical microbial exposure needs to be. Must exposure be sufficient to cause clinical infection or at least asymptomatic colonization, or is ‘subclinical exposure’ adequate to produce the required immune response? The evidence (see review by Beumer et al. [189] suggests that, for some pathogens, the ‘infectious dose’ may be as little as 1–100 cells or particles, while for others, exposure to several thousands of units may be required to elicit clinical infection.

#### The impact of cleaning and hygiene practices on cross contamination and microbial exposure in the home

When people set about ‘cleaning’ their home, or their hands, or body surface, visual observation is used to decide whether ‘cleanliness’ has been achieved. The assumption that visibly clean means ‘free from microbes’ is a misconception. In practice a hygienically clean surface, i.e a surface free from microbes, can be achieved using soap or detergent and water, but, as this involves mechanical removal of the microbes, to be effective it must be properly applied, and used in conjunction with a thorough rinsing process under running water. The alternative method is to apply a disinfectant product which kills the microbes *in situ*.

A range of ‘in-homes’ studies, as reviewed by Beumer et al. [189] demonstrate that soap or detergent-based cleaning routines, as they are currently practised in the home, may have only a limited effect in reducing exposure to microbes. Thus for example, Cogan et al. [200] demonstrated that, following preparation of *Salmonella-* and *Campylobacter-*contaminated chickens in domestic kitchens, 15.3% of hands, and hand and food contact surfaces still showed evidence of contamination even after participants had carried out a washing-up routine with detergent and hot water in a washing-up bowl and then used the cloth to wipe surfaces ‘clean’. A separate study involving the hands, cloths and chopping board showed that, where surfaces were cleaned using the bowl-wash routine but then rinsed under running water, a more significant reduction was achieved, but 23% of 60 sites sampled were still contaminated with *Salmonella*. A recent study [218] showed that, where surfaces contaminated with a faecal suspension infected with norovirus were cleaned using detergent solution applied with a cloth, the virus was not eliminated from the surface. Detergent-based cleaning was insufficient even where the cloth was rinsed in clean water and the surface re-wiped. These and other studies [200, 218, 221] clearly demonstrate that, where a hygiene procedure fails to eliminate contamination from a surface and the cleaning cloth or mop is then used to wipe another surface, the contamination is transferred to that surface, and to the hands of the person handling the cloth.

Preventing microbial transfer in the home depends not only on the effectiveness of the hygiene procedure, but also on when it is applied. The critical influence of these factors on microbial exposure is rarely appreciated. An ‘in homes’ study to evaluate the effects of routine day-to-day home cleaning [222], showed that, before cleaning, one in five (20%) of 10 selected hand and food contact and other sites in the kitchen, bathroom and toilet could be considered as hygienically clean (<10 colony forming units per 25 cm^2^). After detergent-based cleaning, the proportion of contaminated sites was actually increased to 68%. Although disinfectant products were effective in reducing microbial contamination levels, the effects were relatively short lived. After a relatively limited period (90 min to 3 h), most sites and surfaces become substantially re-contaminated. This is probably because of reuse, redisposition from the air or, for wet sites or surfaces (e.g. for damp cloths), regrowth of residual survivors not destroyed by the hygiene process. In other studies where effects were monitored over longer periods (3 days to 9 months [211, 222] the authors concluded that casual use of disinfectant cleaners for daily or weekly cleaning is unlikely to reduce the risk of exposure to pathogens.

The ineffectiveness of non-specific routine cleaning activities in reducing infection exposure is supported by a recent study of home hygiene practices and infection in 398 households of an inner city population [223]. Only two specific practices, using a communal laundry and not using bleach in communal laundering, were found to be predictive of increased risk of infection. Other general cleaning practices such as daily personal bathing or showering, daily cleaning of bathrooms and toilets, frequent changing of dish-sponges, or use/non use of antimicrobial cleaning products for these activities, showed no significant correlation with infection rates.

In arguing the proposition that reduced exposure to microbial pathogens has resulted from changes in domestic cleaning and hygiene practices in recent years, this begs an assumption that patterns of hygiene behaviour in the home are of a type and quality that reduces pathogen exposure. Although there is good evidence to show that handwashing and other hygiene interventions in the home can have a significant impact in reducing the incidence of infection [224] a number of observational studies suggest that compliance with hygiene practices that specifically protect us from pathogen exposure is relatively poor. A study of 108 UK participants to estimate the risk of food poisoning following domestic food preparation showed that only a small proportion of consumers (4.6%) fully implemented appropriate food safety measures while 3.7% prepared food in a way that seriously violated recommended practice and exposed them to a high level of risk [225]. These results complement the studies of Cogan et al. [200, 226] described previously, which showed the extent to which pathogens are spread, from a contaminated chicken during food preparation in a domestic kitchen. Similarly Curtis et al. [227] studied the hygiene practices of mothers with young children who had recently been vaccinated for polio, and were consequently shedding the virus in their faeces. Only 43% of childcarers washed their hands after changing a nappy compared with 76% who washed them after toilet visits. Nappy changing took place mainly in the living room and contact with living room surfaces and objects during nappy changing was frequent. Not surprisingly, poliovirus was detected on 12% of living room surfaces, 10% of kitchen surfaces and 15% of bathroom sites. Hand contact sites were most frequently contaminated, such as bathroom taps, toilet flushes and door handles, soap dispensers and nappy changing equipment.

### Discussion of the hygiene hypothesis and the implications for hygiene practice

The link between atopy, and microbial exposure and infection

In the first part of this paper the evidence for a causal link between the sharp rise in atopy over the past 30 years and the possibility of a reduction in our level of exposure to microbes was reviewed. Although many of the studies cited in support of the hygiene hypothesis are based on proxy measures of microbial exposure, some provide striking evidence supporting such a link. A consistent finding is the inverse relation between atopy, family size and birth order. There is also an apparent protective effect for children living on a farm. In addition however, there are numerous contradictory studies, and the totality of the evidence remains inconclusive.

Some proponents of the hygiene hypothesis suggest that the infection exposure necessary for immune priming should be ‘intense’ [228] or at least produce clinical disease [69]. In this review, data covering the past 100 years was examined in order to look for infection trends which might correlate inversely with trends in atopy, supporting the hypothesis, and might provide clues as to the nature of the critical exposure. The data show that the decline in serious infections such as cholera and typhoid, mumps, rheumatic fever and tuberculosis occurred too early to be associated with the rise in atopic disease in the late 20th century, unless the mechanisms are such that the effects are manifest only in subsequent generations. There is conflicting evidence regarding an inverse relationship between atopy and exposure to infections such as measles, HAV and *Mycobacterium tuberculosis*. While ‘old’ infections with high mortality have declined, this has, to an extent, been offset by the emergence of new infections with high mortality, or those which have re-surfaced (e.g. tuberculosis). Introduction of measures designed to reduce the burden of ID, such as improved housing, sanitation and clean drinking water, correlate with the decline in life threatening enteric disease during the first part of the 20th century, rather than the later rise in atopy. Reduced consumption of food-borne pathogens is also an unlikely candidate as the incidence of food poisoning rose during the critical period of the rise in atopic disorders.

Intuitively the idea that exposure to invasive infection, with all the attendant risks might be needed to protect against atopy seems inefficient in evolutionary terms. A more plausible explanation is that the critical change involves less severe endemic infections. As far as ID morbidity is concerned, there is no evidence of a general decline across the broad range of gastro-intestinal, respiratory and other endemic infections, even in developed countries. Additionally, although the findings of a recent large scale study of 24 341 mother–child pairs [229] confirmed that larger family sizes, early childcare, pet keeping and farm living correlates with decreased risk of atopic dermatitis (AD) in children before 18 months, the results suggested that experience of ID in early life is associated with increased, rather than reduced, risk of AD.

An alternative possibility is that, rather than infection, ‘background’ exposure to ‘subclinical’ doses of pathogens, or to commensal or environmental microbes, particularly those with low invasiveness or virulence such as the rapid growing saprophytic strains of *Mycobacteria*, or perhaps to endotoxins could be the key. In a recent review paper, Rook [13] proposes that the immune dysregulation associated with increased risk of atopy is a consequence of decreased exposure to certain microbes that are ‘old friends’, because of their continuous presence throughout mammalian evolution. He proposes that organisms such as saprophytic mycobacteria, helminths and lactobacilli are recognized by the immune system as harmless, and act as adjuvants for immune regulation. This has prompted work on the development of mycobacterial vaccines for the treatment of some diseases associated with immune dysregulation, with promising initial results [116, 117]. The apparent protective effect against atopy of living on a farm in childhood is consistent with the possibility that ‘background exposure’ may be the critical factor.

Thus, despite some good evidence supporting a link between microbial exposure and susceptibility to atopic disease, clear evidence is still lacking as to the nature of the critical changes that might have occurred, whether it is the general level of exposure which is important, or exposure to specific microbes, whether exposure is only important at certain times of life, or whether the route of exposure is important etc.

The link between atopy, microbial exposure and hygiene practice in the home

The second key question for this review is whether the microbial exposure that is vital for the development of the immune system might no longer occur, or might occur to an insufficient extent, is a result of modern trends in hygiene and personal cleanliness.

Evidence of a link between atopy and domestic cleaning and hygiene is weak at best. Data published since the 1980s, as reviewed in this paper, show that our modern homes, whatever their visual appearance, still abound with a rich mixture of bacteria, viruses, fungi and moulds, as well as dust mites and other insects, and that our opportunities for exposure to these are quite likely to have increased rather than decreased, since a rising proportion of time is spent indoors [5]. The evidence shows that human, animal and food-borne microbes are continuously brought into the home and that transmission from these and other sources via hands, hand contact surfaces, food preparation surfaces and cloths during normal daily activities provide ample opportunities for exposure to foodborne pathogens or pathogens from infected people or pets, as well as exposure to commensals and environmental microbes.

Although consumption of cleaning products has increased over time, consumption overall or for individual product types for individual European countries shows no correlation with levels of atopy. In reality routine daily or weekly cleaning habits actually have little effect in reducing exposure to microbes beyond the levels that have probably prevailed throughout the rise in atopy, even where they involve use of a disinfectant. Re-colonization of surfaces rapidly occurs and many species are adapted to survival, even on apparently dry surfaces. Contrary to perception, domestic cleaning practices can actually increase the distribution of microbes in the home. While ‘hygiene’ practice (i.e. the specific actions we take to prevent transmission of disease) has been shown to be associated with reduced infection rates, observational studies indicate that consumer adherence to basic hygiene rules remains poor. Although the pattern of microbial exposure in the home may have changed, there is no evidence that our modern preoccupation for cleanliness has resulted in a decline in overall microbial exposure.

The suggestion that trends towards more frequent showering and bathing show a temporal correlation with the rise in atopy is superficially consistent with the results of the ALSPAC Study [184, 185], but requires further investigation. In this study the emphasis was towards ‘routine’ rather than ‘targeted’ hygiene i.e. ‘parents were asked how often in a day they wiped the child's face and hands, whether hands were wiped before meals and how often the child was given a bath or shower’. Only 0.4% of children had the highest hygiene score, an insufficient proportion to account for the several fold rise in atopy that has been seen across the whole population.

From the evidence linking atopy to declining family size, it can be argued that, regardless of hygiene behaviour, a decrease in the number of people in the home inevitably decreases opportunities for person-to-person transfer of human commensals, or case-to-case spread of infections via direct or indirect contact or airborne transmission, although much depends on socio-economic factors such as overcrowding, bed-sharing and education level. However, if exposure to childhood infections or commensals is important, it should be found that the effects of decline in family size are offset by increased opportunities for exposure resulting from attendance at day nursery. Although there is some supporting data, other studies show no evidence of a protective effect. The strong evidence for a link between farm living and reduced risk of atopy also supports the possibility that exposure to environmental microbes (and possibly helminths) in our outdoor environment could be the key factor.

Quite apart from hygiene, there are a number of other lifestyle, medical and public health trends which could equally well have caused incidental changes in microbial exposure, manifesting as increased risk of atopy. For example, changes must have occurred in the non-pathogenic microbial flora of water or foods consequent on changing technologies of water purification and food production etc, but as food and water is only routinely monitored for pathogen content there are no data to show what these trends might have been. Alternatively the changes may have been generated by the introduction of antibiotics. This might have operated either by changing the nature, intensity and duration of exposure to pathogens, or by altering the normal balance of commensal microbes such as the gut flora. Although this fits well with the rise in atopy in temporal terms, the supporting data is inconsistent. The balance of evidence is also against vaccination as a causative factor. More important perhaps is the significant evidence supporting a range of ‘non-microbial’ factors, such as diet, obesity and lack of exercise which may be causative factors in the rise in atopy.

The implications for hygiene practice

On the basis of current evidence, relaxing hygiene standards seems neither justified, nor rational. On the contrary, current concerns about ID, and the key role that hygiene plays in controlling ID, provides compelling reasons why we should not do this.

As discussed previously, the global burden of ID is still a major concern, accounting for over 18 million deaths annually: while the majority of deaths occur in the developing world, infection also causes around 4% of deaths in developed countries [230]. Although ID mortality is declining in the developed world, trends in morbidity suggest a change in the pattern of ID rather than declining rates. This is partly associated with emergence of new infections, such as *E. coli* O157 and the re-emergence of old pathogens; tuberculosis has increased in Europe, including more invasive and antibiotic-resistant strains, while in the former soviet block countries, diphtheria cases rose 50-fold from 1989 to 1995 [230]. The globalization of infection risks has also increased because of global food markets, increased travel and refugee movements: thus pathogens can more readily reach areas where there is little or no innate resistance:

In recent decades, our attitudes to ID in developed countries have become more relaxed, nurtured by the evidence that improved quality of water, sanitation, housing and nutrition have produced a marked decline in infection mortality from infections such as typhoid fever and cholera. Antimicrobial therapy and advances in immunization have supplemented this trend. Yet several factors are combining to make it likely that the threat of ID will increase in coming years, rather than decrease. One such factor is the rising proportion of the population who are more vulnerable to infection. Such ‘vulnerable groups’ include the elderly: it is estimated that, by 2025, more than 800 million people will be aged over 65 years in the world, two-thirds of these in developing countries [231]. Other groups include neonates, people with chronic or degenerative illness; and immuno-compromised patients discharged from hospital. All of these groups, together with family members who are carriers of HIV, are increasingly cared for at home. Currently, about one in six persons in the UK belongs to an ‘at risk’ group, and it is likely that the same applies in most European countries [232].

In addition to the threat posed by acute infections, pathogens are increasingly implicated as causative or as co-factors in cancers and some degenerative diseases. Examples include Hepatitis B virus [233], *C. jejuni* [234] and *H. pylori* [235]. Foodborne illness has been estimated to result in chronic sequelae in 2–3% of cases [236]; a more recent report from the European Commission [237] cited evidence of chronic disease, such as reactive arthritis, following 5% of *Salmonella* cases, with 5% also of *E. coli* O157 cases progressing to the serious and often fatal complication of uraemic syndrome.

Antibiotic resistance is a global problem with resistant strains such as MRSA spreading into communities [238, 239]. The need for improved hygiene to reduce the spread of antibiotic resistance has been recommended by recent working parties in Europe [240]. Reduced rates of infection and antibacterial resistance have been demonstrated where an approach combining good hygiene and reduced prescribing has been evaluated [241, 242].

Measures such as water purification, sanitation, waste disposal and hygiene have played an essential role over the past two centuries in reducing the burden of ID, most markedly in developed countries. For developing countries, where the ID burden remains high, it is now apparent that health gains commensurate with investment in programmes of water and sanitation will only be achieved if steps are also taken to improve standards of hygiene practice. A review of intervention studies [224] suggests that, even in the developed world, improved standards of personal and environmental hygiene could reduce infection transmission by over 20%. The overall conclusion is that hygiene is a key cornerstone in the control of ID, and that a significant increase in morbidity and mortality from infection would result from any attempt to reduce the integrated practices of sanitation, clean water provision and hygiene practice.

### Developing a rational approach to home hygiene

Regardless of whether the hygiene hypothesis is correct, the popular interpretation that ‘dirt is good for us’ [243] has considerably influenced attitudes, and caused loss of confidence among the public regarding home hygiene. One positive benefit however is a recognition by public health professionals of the need to provide clearer guidance. One of the concepts which we need to clarify is the difference between ‘dirt’ and ‘germs’, and between ‘cleanliness’ and ‘hygiene’. Without knowing the nature of the microbial exposure which may be critical for immune priming, it is difficult to reformulate hygiene policy, in favour of improving immune function without compromising protection against ID. Even if we had the correct information, selective targeting of hygiene interventions, as a means of maintaining beneficial microbial exposure, would only be an option if their mode of transmission were significantly different from that for pathogens. If it were proved that intense infection is an essential factor, the evidence suggests that encouraging such exposures would cause significant morbidity and mortality; if the main effect was, for example, a reduction in hayfever, with little or no impact on asthma, the ‘trade off’ would represent a very poor bargain. If it turns out that more general ‘background’ exposure is needed, e.g. organisms with low invasiveness or virulence, such as the rapid growing saprophytic strains of *Mycobacteria*, the idea of ‘right’ and ‘wrong’ types of microbial exposure is academic, unless we could engineer the ‘right’ exposure, without introducing dangerous organisms. As untreated water may contain up to 10^9^ mycobacteria per litre, the difficulty is how to preserve the ‘friendly’ species while removing those likely to cause disease. One option that is already being pursued is an attenuated vaccine containing the ‘right’ type of microbes (e.g. saprophytic mycobacteria), and there is evidence of efficacy of a vaccine strategy in animal studies [244] and in some of human trials [245, 246]. With vaccine strategies, there is no conflict with hygiene.

If background exposure is proved to be the important factor, this opens up the way to promote an approach to hygiene which focuses on preventing exposure to infectious doses of pathogens, but is more relaxed about other exposures. As part of its work to promote better understanding of hygiene and better hygiene practice, the International Scientific Forum on Home Hygiene (IFH) has produced guidance documents on home hygiene practice [247, 248]. The key feature of the guidelines is that they are based on the concept of risk assessment and risk prevention. [249, 250]. Risk assessment (also known as HACCP or Hazard Analysis Critical Control Point) is now the accepted approach for controlling microbial risks in food and other manufacturing environments. Applied to the home this has come to be known as ‘targeted hygiene’.

The IFH guidelines start from the premise that homes always contain potentially harmful microbes (from people, pets, food, etc.) and that ID prevention is not about eradication, but about targeting measures in the places and at the times that matter, in order to limit risks of exposure. Fundamental to developing infection prevention policies is the need to recognize that the home is an environment where all human activities occur including food hygiene, personal hygiene (particularly hands) and hygiene related to care of vulnerable groups, all of which are based on the same underlying microbiological principles. Hygienic cleanliness (reducing the level of contamination to a level that does not pose a significant risk) is required only where the infection risk is significant e.g. after contact with excreta, during food preparation etc. The level of risk varies according to occupants of the home (e.g. presence of children, pets, ill people) and their immune status.

Although this review concludes that the relationship of the hygiene hypothesis to hygiene practice has not been proved, it lends strong support to initiatives which seek to improve hygiene practice. Whatever the reality regarding atopy and microbial exposure, ‘targeted hygiene’ with its emphasis on selective hygiene intervention when and where risks of infection are greatest makes sense on its own merits because it seeks to maximize protection against the harmful effects of ID, while retaining the beneficial effects which microbes may have on our human and natural environment.

The term ‘hygiene hypothesis’ initially raised awareness of the role of microbes and their products in immune regulation. It has also stimulated a considerable amount of research into the aetiology of atopic disease. This research has highlighted that the term has probably now outlived its usefulness. Several of its proponents have suggested renaming the hypothesis as the ‘microbial deprivation hypothesis’ (Bjorksten [251]) or the ‘old friends hypothesis’ (Rook [13]). Avoiding the term ‘hygiene’ would help focus attention on determining the true impact of microbes on atopic diseases, while minimizing risks of discouraging good hygiene practice.
